# Description and mid-term results of the ‘over the top’ technique for the treatment of the pincer deformity in femoroacetabular impingement

**DOI:** 10.1093/jhps/hnv058

**Published:** 2015-08-14

**Authors:** Victor M. Ilizaliturri, Pedro Joachin, Marco Acuna

**Affiliations:** 1. Department of Adult Joint Reconstruction, National Rehabilitation Institute of Mexico, Av. México-Xochimilco No. 289, Col. Arenal de Guadalupe, Mexico D.F., C.P. 14389, Mexico

## Abstract

Pincer impingement is often treated by surgical labral separation from the acetabular rim and rim reduction. A more recent technique the so-called ‘over the top’ involves reduction of the bony acetabular rim without separation of the labrum. Our purpose is to report mid-term results of the ‘over the top’ technique. Between January 2006 and January 2013 a consecutive series of patients with femoroacetabular impingement (FAI) diagnosis, treated with the ‘over the top’ technique were included, using the lateral approach. The Western Ontario and MacMaster (WOMAC) scores were evaluated. Fifty patients (20 males and 30 females) from the Hip and Knee Joint Reconstructive and hip arthroscopy division were included. The average age was 30.5 years old and the average follow-up was 48 months (range 70–90). Preoperative WOMAC average was 42. Post-operative WOMAC was 81.3 (*P* = 0.01). One patient required an arthroscopic revision due to adherences, but had a full recovery after the revision surgery. The ‘over the top’ technique is an excellent choice for the treatment of the pincer deformity in the FAI avoiding the injury of the chondrolabral union.

## INTRODUCTION

The concept of femoroacetabular impingement (FAI) has evolved considerably and is now accepted as an important factor related to hip pain and a probable cause of hip osteoarthritis [1]. The first described treatment was proposed by Ganz *et al.* [2] and consisted of a controlled open surgical dislocation and remodeling of the impinging deformities with good initial clinical results. Over the past decade, hip arthroscopy has been recognized as a standard procedure in the treatment of FAI [3]. Recent literature compares open versus arthroscopic treatment [4, 5], concluding that both were associated with clinical improvement. Botser *et al.* [5] suggest that hip arthroscopy is associated with a faster recovery and less complications. Mid-term results for arthroscopic treatment of FAI have been reported ranging from good to excellent [6, 7].

 Pincer type FAI may be treated with labral debridement and trimming of the acetabular over-coverage, resulting in relief of symptoms [8, 9]. However, the labral function could be compromised because of partial resection [10]. Philippon *et al.* [11] in an ovine model demonstrated that, when the chondrolabral junction was surgically disrupted, the labrum can be reattached with a suture anchor and a single suture, healing occurs subsequently by a fibrovascular scar from the capsule and the burred bone surface at the acetabular rim after 12 weeks. Based on the evidence that the labrum may heal [11], the technique of detaching the labrum to expose and reduce the bony acetabular rim therefore correcting the pincer and subsequently reattaching the labrum with suture anchors was introduced [12, 13, 14]. Sampson *et al.* [15] suggested an acetabular bone resection, behind the intact labrum, without separating the labral chondral junction. Syed and Martin [16] reported good-to-excellent results in 84% of their series of 145 pincer remodeling cases with a minimum follow-up of 2 years.

The purpose of this study is to describe and report our results using ‘over the top’ technique (rim remodeling without chondral-labral separation) for the treatment of Pincer type FAI. Our hypothesis was that remodeling of a pincer lesion with labral chondral junction preservation is a reproducible surgical technique with good-to-excellent clinical outcomes at a mid-term follow-up.

## MATERIALS AND METHODS

**Fig. 1.**
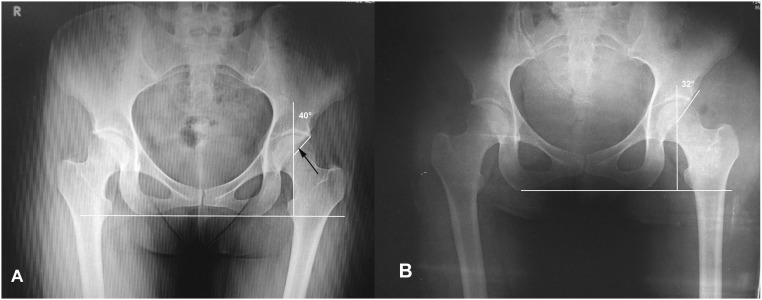
**A.** Preoperative AP pelvis of a mixed FAI deformity in a left hip. The CE angle is 40°. The black arrow points to the inferior limit of a ‘crossover sign’. **B.** Three years follow-up AP pelvis (at 3 years) showing preserved joint space. A CE angle of 32° and the absence of a ‘crossover sign’.

**Fig. 2.**
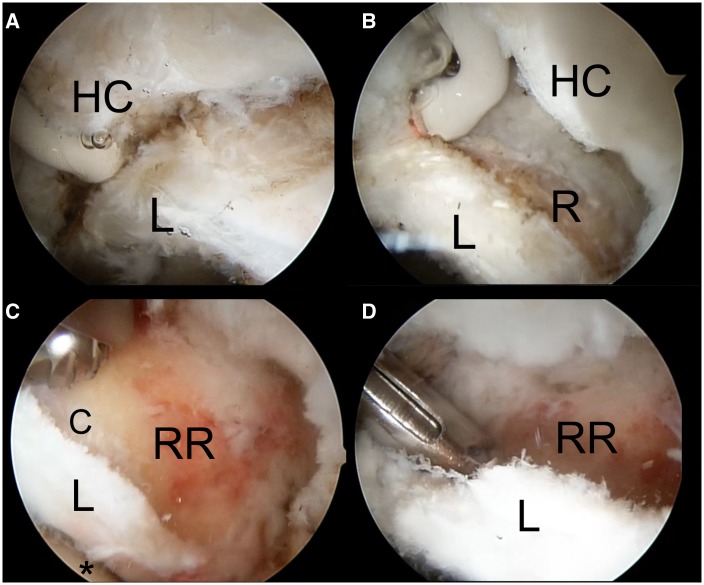
Arthroscopic sequence of pincer remodeling in a right hip. **A.** This image demonstrates the process of elevating the hip capsule (HC) from the acetabular rim proximal to the labrum (L) using a radiofrequency (RF) probe as visualized from the direct anterior portal (the RF probe is introduced through the anterolateral portal). **B.** The acetabular rim (R) has been exposed proximal to the labrum (L), the condral labral junction was not separated. The hip capsule (HC) is observed at the right. **C.** The remodeled acetabular rim (RR) is at the center of the photograph. The articular cartilage (C) was exposed after the overhang of the pincer deformity was removed. The labrum (L) is observed in continuity to the articular cartilage (C). A 5.5-mm burr is at the top of the photograph. **D.** A suture anchor is being introduced on the resected acetabular rim (RR) to reinforce the condral labral junction.

**Fig. 3.**
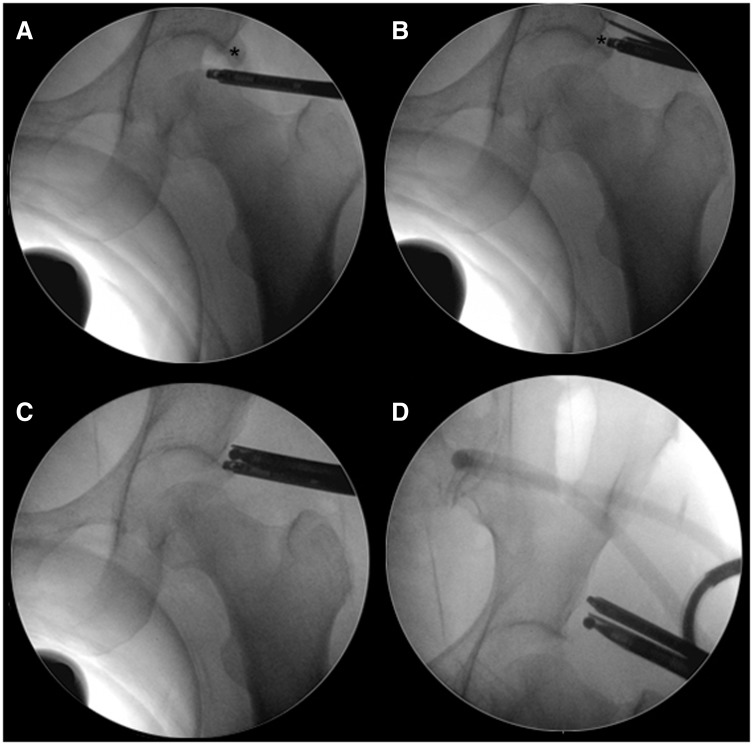
Series of fluoroscopic images of a left hip during the process of pincer remodeling. **A.** The hip is with traction. A 70° arthroscope is position in the central compartment. Asterisk indicates the pincer deformity. **B.** A 70° arthroscope is visualizing the pincer deformity (asterisk). A radiofrequency probe is used to elevate the hip capsule proximal to the pincer deformity (asterisk). **C.** A burr is used to remodel the pincer deformity under direct arthroscopic vision. **D.** End result of rim remodeling.

**Fig. 4.**
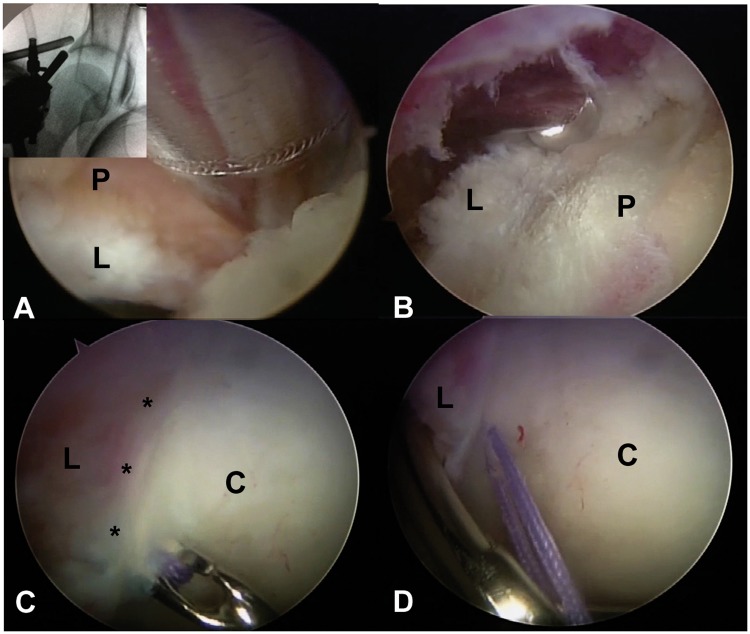
Sequence of photographs demonstrating pincer re-shaping in a right hip. **A.** A shaver is used to remove tissue from the pincer (P) overhang as viewed from the capsular side. The labrum (L) is at the bottom of the photograph. Fluoroscopic image demonstrates the position of the shaver and the arthroscope. **B.** A spherical burr is used to re-shape the pincer (P) deformity. The labrum (L) is to the left of the photograph. The view is from the ‘over the top’ position. **C.** Intra-articular view of the chondral (C) labral (L) junction. The asterisk demonstrates integrity of the chondral-labral junction. A suture passer is used to pass a suture. Note how the enter point is medial to the chondral–labral junction because the pincer deformity has been removed. **D.** The suture is retrieved with a piercing technique through the labrum (L). The acetabular cartilage is to the left (C).

We report a consecutive series of patients that were treated with hip arthroscopy for pincer or mixed type FAI, using rim reduction without separation of the chondrolabral junction. Diagnosis was based on clinical findings, anteroposterior (AP) pelvis and frog leg lateral hip radiographs. Magnetic resonance arthrograms were performed in every case. All the patients that presented pincer FAI were detected by the ‘crossover sign’ (anterior wall overlapping over the posterior wall on the AP pelvis), observed at the AP pelvis radiographs (Fig. 1). Inclusion criteria were aged from 15 to 60 years old, pincer or mix impingement and a Tonnis 1 score in AP pelvis. Patients with existing chondral–labral disruption found at arthroscopy due to impingement were not included. All the patients were operated in lateral decubitus, traction was used to access the central compartment and was removed for examination of the peripheral compartment and dynamic evaluation of impingement. A capsulotomy between the anterolateral and direct anterior portal was performed in every case (the capsule was not repaired). The hip capsule was elevated proximal to the labrum at the perilabral junction using radiofrequency (Fig. 2), fluoroscopy was used to confirm adequate position on the capsular side of the pincer deformity and throughout the pincer re-shaping (Fig. 3). Once the pincer deformity was exposed a 5.5-mm spherical burr was used for remodeling of the acetabular rim from the capsular side without separating or disrupting the chondral labral junction. One or two suture anchors were used to stabilize the chondral labral complex to the remodeled rim (Fig. 4).

This was performed at the lateral and anterior aspects of the rim (Zones 3 and 2 of the geographic zone method) [17]. The stability of the labrum and effectiveness of the labral seal on the femoral head were assessed dynamically without traction.

Cam type deformities were treated by arthroscopic remodeling through the same capsulotomy (between the anterolateral and direct anterior portals) under direct arthroscopic vision dynamic assessment and fluoroscopic navigation.

The senior author performed all arthroscopic procedures.

The patients were evaluated with an inverted polarity Western Ontario and McMaster Universities (WOMAC) osteoarthritis index (in Spanish), preoperatively and in the last follow-up.

CE angle was measured preoperative and post-operative with AP pelvis radiographs (Fig. 1).

## RESULTS

Between January 2006 and September 2013 a consecutive series of 50 patients that met the inclusion criteria were studied and prospectively followed (20 males and 30 females). Thirty-four hips were right and 16 were left, 29 had pincer type and 21 had mixed type impingement. The average age was 30.86 years old (range 15–58) and the mean follow-up was 41.24 months (range 12–62). Statistical analysis was performed with the SPSS software version 15.0 (SPSS Inc., Chicago, IL). Preoperative WOMAC average was 43.32 ± 11.67. Post-operative WOMAC was 82.74 ± 5.09. The comparison between both results was statistical significant (*P* = 0.01). Preoperative CE angle was 28.41° and post-operative 25.75°, with and average rimming angle of 2.97°. The mean rim reduction was 4 mm (range 1–6 mm). In 23 patients only one lateral anchor was used (zone 3), 27 patients required a lateral and an anterior anchor (zones 3 and 2). There were no patients that required only anterior anchors (zone 2). One patient required an arthroscopic revision due to adherences., The chondral labral junction was found stable at revision surgery and no further fixation or bone resection were needed and had a full recovery after the second procedure.

## DISCUSSION

This study shows a novel technique proposed to reinforce the chondral labral junction after pincer treatment without chondral labral separation. Gedouin *et al*. [8] presented good results with labrum detachment and reattachment, measured also with the WOMAC score. In our study the chondrolabral union was preserved, with the biological advantage of preserving the cellular continuity of the chondral and labrum junction and the mechanical stability of an uninterrupted labral insertion to the articular cartilage. Syed and Martin [16] reported good-to-excellent results without detaching the labral chondral junction. Our results showed a significant clinical improvement according to the WOMAC scores when the labrum is not detached at all. Philippon *et al.* [18] found in patients with arthroscopic remodeling of pincer deformities that the CE angle in AP pelvis radiographs preoperatively was 36.4° and postoperatively 32.3 (3.9° in average), having similar results with ours, that was an average 2.9° CE change and a 4-mm average reduction.

### Limitations

This is a report on a prospective and consecutive series of patients treated for a pincer or mixed impingement deformity without chondral labral separation. A randomized study comparing labral takedown pincer remodeling versus chondral labral preservation would provide a higher level of evidence on this subject.

## CONCLUSIONS

On the basis of the findings of our study, we considered that the ‘over the top’ technique provides good to excellent results for the treatment of the Pincer and mixed deformities in FAI.

## CONFLICT OF INTEREST STATEMENT

None declared.
